# M. Roux on Fistula of the Parotid Duct

**Published:** 1829-10-01

**Authors:** 


### Successful Mode of treating Fistula of the Parotid Duct. By M. Roux.*

A salivary fistula is neither a very pleasant nor tractable complaint, and any mode of treatment which shall prove more successful than those in common use, will undoubtedly be a boon to surgery and surgeons. We have no intention of entering into prolix details respecting fistulæ of the duct of the parotid gland, or duct of Steno, as it is usually termed; we would simply remark that it is rarely met with, and is one of the most obstinate of the salivary fistulæ. When it opens within the mouth, through the mucous membrane lining the cheek, it can scarcely be called a disease, as the fluid, in that case, is discharged, though not by its original route, into its natural receptacle. But when the opening is situate externally, and the saliva, instead of reaching the mouth, dribbles down the cheek, a train of inconveniences arise, which finally fall under the cognizance of the surgeon, and which it is his interest and business to remedy, if he can. The following case, which occurred in the private practice of M. Roux, will shew that eminent operator's methodus medendi.

M. Auguste Godin, a notary at Neuvy, was loading a fowling-piece, when his powder-flask exploded, and one of the fragments produced a wound in the right cheek. The wound was both deep and wide, and being in the situation of the parotid duct, great pains were taken to procure union by the first intention. Much inflammation, however, succeeded, and the above object was but partially accomplished; suppuration succeeded; and, along with the pus, saliva flowed in considerable quantity. It was now quite obvious that a salivary fistula was established. Cauterization of the edges of the ulcer, and afterwards compression were employed, but they failed in effecting a cure, and, two months after the accident, M. Godin repaired to Paris and consulted M. Roux. The fistulous opening evidently communicated with the duct of Steno, and not with the substance of the parotid gland. On introducing an Anel's probe through the natural orifice of the duct, on the inside of the cheek, it was stopped, after a course of about half an inch, by what appeared to be an obliteration of the canal, and the same thing took place on attempting to pass the probe from the fistulous opening without.

Finding that the cavity of the duct was obliterated between the fistula and the orifice in the mouth, M. Roux was convinced that neither the caustic, nor compression, nor any other similar proceeding could avail, unless a route into the mouth were made for the saliva. M. Roux accordingly proceeded to an operation for that purpose, and first of all exposed the bottom of the fistula, by excising a portion of diseased skin which surrounded the external opening. The next step consisted in making two successive perforations of the cheek, from without inwards, by a hydrocele trocar, taking care to avoid the gum. The openings were made on a horizontal line, and a quarter of an inch distant from each other; the one corresponded directly with the bottom of the fistula, exactly opposite the external opening—the other was nearer the parotid gland, and close to the anterior border of the masseter muscle. A small seton, composed of silk threads, was then passed through both openings, and the ends tied loosely in a knot, and retained on the outside of the cheek.

---

* Journal Hebdomadaire, No. 18.

A good deal of swelling and inflammation of the cheek succeeded, but were quickly subdued by local applications. A superficial abscess, however, formed close to the parotid gland, and communicated with the wound which had been made in the operation. It was freely opened, and the skin, or rather the old cicatrix over it was removed, so that the two wounds were laid into one. The saliva continued for some time to flow, in part externally, and in part by the openings into the mouth. Light dressings and gentle compression were employed, and the seton-threads were frequently moved backwards and forwards, in order to render the perforations completely fistulous. At one time strong compression was made on the external wound, but it brought on a return of inflammation, which extended to the parotid gland itself; it was, therefore, abandoned. Six weeks after the performance of the operation, the saliva all at once ceased to flow from the wound, which rapidly cicatrized, and soon afterwards the silk thread came away spontaneously. M. G. stopped some time longer in Paris, and the cicatrix which ensued proved to be smooth, regular, and unaccompanied with any appreciable deformity. On the internal membrane of the cheek a transverse sulcus, with slightly elevated margins, could be felt, from which the saliva flowed into the mouth.

We have thought it right to publish the heads of the case, because they display the practice of M. Roux in this troublesome disease. That it ultimately succeeded there cannot be a doubt, but that some other method would not have done as well, perhaps there may. We do not clearly perceive the necessity for establishing two perforations in the mucous membrane of the cheek; indeed, we are convinced that one, of sufficiently ample dimensions, would be simpler and better. The seton thread passed through the two openings, and, including a portion of intervening soft parts, must frequently cut through that portion by ulceration, and lay the openings into one at last; or, at all events, if even they remain distinct, the two can answer no useful purpose, that would not be equally or better answered by a single one. This however, is a trifle, and does not affect the principle of the operation, viz. the establishment of an artificial route for the saliva into the mouth. If this be the indication, as it certainly is, it must matter little how the minor steps of the operation that fulfils it are conducted.

